# Synthesis of Fe/Ni Bimetallic Nanoparticles and Application to the Catalytic Removal of Nitrates from Water

**DOI:** 10.3390/nano9081130

**Published:** 2019-08-06

**Authors:** Gunay G. Valiyeva, Irene Bavasso, Luca Di Palma, Sevinj R. Hajiyeva, Mahammadali A. Ramazanov, Flora V. Hajiyeva

**Affiliations:** 1 Department of Ecological Chemistry, Faculty of Ecology and Soil Science, Baku State University, Baku AZ1000, Azerbaijan; 2 Department of Chemical Engineering Materials & Environment, Sapienza University of Rome, Via Eudossiana, 00184 Rome, Italy; 3 Department of Chemical Physics of Nanomaterials, Faculty of Physics, Baku State University, Baku AZ1000, Azerbaijan

**Keywords:** nitrate, wastewater treatment, bimetallic nanoparticles, kinetics

## Abstract

This work investigated the effectiveness of zerovalent iron and Fe/Ni bimetallic nanoparticles in the treatment of water polluted by a high concentration of nitrates. Nanoparticle synthesis was carried out by a sodium borohydride reduction method in the presence of sodium oleate as a surfactant. The particles were characterized by XRD and SEM. Batch experiments were conducted on water samples contaminated by 300 mg L^−1^ of nitrate. The parameters investigated were the Fe/Ni dosage (0.05, 0.1, 0.2, 0.3, and 0.4 g L^−1^) and the reaction pH (unbuffered; buffered at pH = 3; initial pH = 3, 5, and 10). The results showed that almost complete nitrate removal (>99.8%) was always achieved after 15 min at a concentration of bimetallic nanoparticles higher than 0.2 g L^−1^. The optimization of bimetallic nanoparticle dosage was carried out at a fixed pH. Kinetic study tests were then performed at different temperatures to assess the effect of temperature on the nitrate removal rate. By fixing the pH at acidic values and with an operating temperature of 303 K, nitrates were completely removed after 1 min of treatment.

## 1. Introduction

The growth of industrial and agricultural activities has resulted in increasingly large amounts of wastewater that need a specific treatment before being released back into the environment. Nitrate (NO_3_^−^) is the most diffused contaminant, especially because of its high solubility in water and low retention by soil particles [1]. High NO_3_^−^ contamination is mainly due to agricultural fertilizer application and animal farming in agricultural regions [2], septic tank use, atmospheric deposition, and industrial and wastewater discharges [3].

Nitrate is prone to leaching to subsoil layers and, ultimately, to groundwater if it is not taken up by plants or denitrified to N_2_O and N_2_, and its leaching rate is governed by soil properties and the amount of water present in the soil system [4].

Nitrates are important for plant life as a source of mineral nitrogen [5], but they are toxic for humans because, after their assimilation, they are reduced into nitrites, which react with amines or amino acids to form nitrosamines, which are recognized as carcinogenic and mutagenic agents. In addition, a potential consequence of high nitrite and nitrate concentrations in drinking water is non-Hodgkin’s lymphoma [6]. Nitrate has been implicated in methemoglobinemia and a number of currently inconclusive health issues, including hypertension, damage to the central nervous system, diabetes, birth defects, spontaneous abortions, respiratory tract infections, and changes to the immune system [4,7]

To limit the risk to human health from nitrate in drinking water, the maximum contaminant level (MCL) has been set at 10 mg N L^−1^ (45 mg NO_3_^−^ L^−1^) by US Environmental Protection Agency, while the World Health Organization and the European Community have set the MCL at 11.3 mg N L^−1^ (50 mg NO_3_^−^ L^−1^) [7].

Various techniques such as ion exchange, reverse osmosis, and electrodialysis have proved to be highly effective at removing nitrate contaminants, but their implementation in large-scale water treatment plants is often limited by their high energy consumption, technological complexity, nitrate brine concentration, and secondary pollution [8]. While reverse osmosis can treat more than 90% of nitrates, the equipment and energy costs for operation are very high. This method is also a very slow process; sometimes treatment can take a few days, but as shown in the literature, 98% nitrate removal was achieved only after 839 h of treatment [9]. Initial nitrate concentration in water membrane fouling may also have a negative impact on nitrate removal. As previously reported, for initial nitrate concentrations of 25 and 200 mg L^−1^, nitrate removal was 93.5% and 82.5%, respectively [10]. Biological denitrification is the most widely applied process, though it is quite slow, difficult to control, has high monitoring needs, and it is inhibited by nitrite formation, especially when the nitrate concentration in wastewater is higher than 100 mg L^−1^ [11,12].

Chemical reduction of nitrate is of growing interest because it degrades nitrate faster than biological methods and is more cost effective than many physicochemical techniques [13]. Nitrate removal efficiency depends on several factors, such as pH, temperature, contact time, the concentration of the nanoparticles used for the treatment, and the initial concentration of nitrates [14]. Several metal reductants, including magnesium (Mg), nanoalumina (Al), and zerovalent iron (nZVI), have been tested for nitrate treatment. For example, nitrate removal by 2 g L^−1^ of zerovalent magnesium at pH 2 was 93%, with a contact time of 20 min and at an initial nitrate concentration of 50 mg L^−1^ [15]. Nitrate removal efficiency by zerovalent aluminum also depends on several parameters: the optimal conditions (nitrate reduction between 40% and 50%) have been assessed at a pH of 9–12, aluminum powder dosages of 40–250 mg L^−1^, initial nitrate concentration of 10–50 mg L^−1^, and reaction time of 5–60 min. However, at an initial nitrate concentration higher than 30 mg N L^−1^, a quick reduction of removal efficiency has been observed, and the main drawback of this reducing agent is the potential water pollution by aluminum [16]. Due to the numerous advantages offered by zerovalent iron nanoparticles, their use in environmental remediation processes is nowadays a common practice [17,18].

Nanosized iron has a larger surface area (about ten to hundreds of times larger) than micro- or millisized iron powder, which enhances its adsorption efficiency [17]. Innovative applications of nZVI for groundwater remediation or the treatment of industrial wastewater contaminated with toxic materials have already been successfully investigated, such as TCE [2], Cr(VI) [18], Pb [19], As (III), As (V) [20,21], and NO_3_^−^ [22,23]. Many researchers have investigated zerovalent iron nanoparticles regarding nitrate remediation from water. Nitrate removal efficiency by nZVI in water has been found to be up to 95% at pH 4, with a contact time of 60 min, an initial nitrate concentration of 50 mg L^−1^, and at a nanoparticle concentration of 15 g L^−1^ [24].

Due to its aggregation behavior and easy oxidation on the surface, nZVI is often coupled to other metals to increase the catalytic reactivity and prevent iron oxidation [25]. nZVI alone, in fact, cannot maintain stable nitrate removal in a continuous treatment process due to the loss of reactivity caused by iron corrosion. It was observed that after 1 h of treatment, the nitrate removal efficiency achieved was 85%, but when the treatment process continued for 7 h, the nitrate removal decreased to 0 [26].

In order to improve nitrate removal, various bimetallic nanoparticles were tested in batch studies. The removal efficiency of Fe/Pt bimetallic nanoparticles at pH 4–10 was between 83% and 92% after 60 min at an initial nitrate concentration of 200 mg L^−1^ [27]. After 20 min of reaction, an almost quantitative nitrate removal was also obtained using Fe/Cu bimetallic nanoparticles [14].

A comparison among selected metals has already been carried out, showing that Pt, Pd, and Cu are the most promising secondary metals to be added to iron nanoparticles [28]. The catalytic effectiveness of noble metals, such as Pd and Pt, was also proved to be higher that Ni [29]. However, Ni is more suitable for environmental applications due to its lower cost, fast reaction rate, and good corrosion stability [29].

In this work, nanoscale bimetallic Fe/Ni particles were synthesized and tested for the treatment of high-strength nitrate-polluted water. Fe/Ni nanoparticles were prepared by chemical reduction from iron chloride hexahydrate [30,31,32].

The removal of nitrates by nZVI and Fe/Ni bimetallic nanoparticles has been already reported in other studies, but reduction tests were generally carried out at the typical nitrate concentration found in domestic wastewater (below 50 mg L^−1^). At such low concentrations, Fe/Ni nanoparticles synthesized by different methods have proved to be very effective, and more than 99% nitrate removal was achieved in about 1 h [32,33]. At higher nitrate concentrations, conventional biological denitrification processes could be ineffective due to the inhibition associated with nitrite formation. Therefore, an alternative to biological processes could be necessary to comply with environmental standards [11,12].

In the present work, the effectiveness of Fe/Ni bimetallic nanoparticles was tested at a high nitrate concentration to assess the influence of the operating conditions on the reaction rate and removal efficiency. The nanoparticles were characterized by XRD and SEM and then used in nitrate reduction tests. The tests were carried out at selected nanoparticle dosages, and the influence of pH on the reaction effectiveness was investigated. The kinetics of the process was investigated, and the reaction mechanism was also investigated by performing mass balances in a closed system.

## 2. Materials and Methods

### 2.1. Chemicals

Iron (III) chloride hexahydrate (PLC 141358, 99% chemically pure), sodium borohydride (PLC 143314, 99% chemically pure), nickel (II) sulphate heptahydrate (PLC 141445, 98% chemically pure), and sodium oleate (C_18_H_33_NaO_2_ PLC 113655) were used for nZVI and bimetallic Fe/Ni particle synthesis. Sodium nitrate (NaNO_3_ LC24650) was used to prepare the nitrate solution. All chemicals were of analytical grade and were used as received without further purification.

### 2.2. Fe/Ni Nanoparticle Production

Bimetallic nanoparticles were synthesized in a flask reactor with three open necks. To avoid agglomeration, dispersing agents were used during synthesis. Several kinds of dispersing agents were studied in this work to prevent iron nanoparticles from aggregating, including sodium oleate (SO), carboxymethylcellulose sodium salt (CMC), cetyltrimethylammonium bromide (CTABr), and polyethylene glycol (PEG). Each of these dispersing agents was brought into contact with three metals (Ni, Cu, and Pd). The most effective combinations were selected based on their rate constants in nitrate reduction reactions, cost effectiveness, and characterization in batch kinetic experiments. It was found that in the presence of a sodium oleate surfactant, bimetallic nanoparticles were more effectively stabilized against oxidation and agglomeration compared with other stabilizers. Of these, SO played a very important role in the dispersibility of the nanoparticles by altering the surface charge distribution, which led to electrostatic stabilization. Thus, the obtained SO–bNP–Fe–Ni nanoparticles were well dispersed in aqueous solution and not prone to agglomeration.

Bimetallic nanoparticles were prepared by mixing 0.1 M Fe^3+^ solution with a 0.5% solution of sodium oleate. It was well mixed on a magnetic stirrer at 500 rpm. After 15 min, 5% of Ni^2+^ was added to the solution. Also, 100 mL of 0.3 M sodium borohydride solution was prepared and added to the above solution dropwise and stirred constantly. The entire reaction was at room temperature and in an oxygen-free atmosphere under nitrogen gas. The mixture was left for another 10 min of stirring after adding the sodium borohydride solution to complete the reaction.

For complete removal of nonreactive ions, synthesized bimetallic nanoparticles were washed three times with absolute ethanol and then used for nitrate removal. A CR4000 Pro-Analytical centrifuge (Centurion Scientific Ltd, Chichester, UK) was used for nanoparticle washing. The morphology of the particles was characterized by SEM (JEOL JSM-7600 F, (JEOL, Tokyo, Japan ) and EDS (Oxford Instruments, AZtecLive, Abingdon, United Kingdom) in SEI mode, with an accelerating voltage of 15 kV and a working distance of 4.5 mm, and by XRD, using a Rigaku Mini Flex 600 XRD instrument (Rigaku, Tokyo, Japan), with Cu Kα radiation from a Cu X-ray tube (run at 15 mA and 30 kV) and a scan area of 20°–80°. The instrument was operated with an accelerating voltage of 30 kV and a current of 15 mA. The nanoparticles were tested at room temperature over the 2θ range with the scan area of 20°–80° using graphite-monochromated Cu Kα radiation. The diffractograms were analyzed using PDXL software (Rigaku, Tokyo, Japan).

pH was measured using a pH meter (PHS-25 C pH).

### 2.3. Nitrate Reduction Experiments

A stock solution of nitrates was prepared by dissolving 0.17 g of NaNO_3_ in 400 mL of distilled and deoxygenated water (nitrate concentration = 300 mg L^−1^.) The batch experiments were performed with bimetallic nanoparticles at different concentrations (0.05, 0.1, 0.2, 0.3, and 0.4 g L^−1^) to test different weight ratios between the nanoparticles and the nitrates (0.1, 0.3, 0.7, 1, and 1.3, respectively).

All reactions were conducted in three-necked flasks at ambient temperature and in an oxygen-free atmosphere under nitrogen gas.

The tests were carried out at the pH of the nitrate solution (pH = 6.5), and the selected initial pH (3, 5, 7, or 10) was adjusted by adding dropwise 1 M H_2_SO_4_ or NaOH.

Additional tests in a closed system were carried out to investigate the process mechanism and byproduct formation. A DR3900 spectrophotometer (Hach TNTplus™) was used to measure the ammonium concentration in water. 

Kinetic tests were performed at selected temperatures (283, 298, 303, and 313 K) and a Fe/Ni nanoparticle dosage equal to 0.2 g L^−1^. Freshly prepared Fe/Ni bimetallic nanoparticles were added to sodium nitrate solution (300 mg L^−1^) in nitrogen atmosphere and stirred for 15 min at 500 rpm. Samples were collected at selected times to investigate the reaction rate (1, 3, 5, 10, and 15 min). The concentrations of nitrate and nitrite were measured by ion chromatography (Dionex ICS-5000). Before injection to ion chromatography, samples were centrifuged on a CR4000 Pro-Analytical centrifuge and filtered by syringe filters (pore size of 0.45 μm) to obtain pure water without nanoparticles. All the tests were performed in triplicate, and a standard deviation lower than 4% was calculated.

## 3. Results

### 3.1. Ni/Fe Nanoparticle Characterization: SEM and XRD Analysis

**Figure 1:**
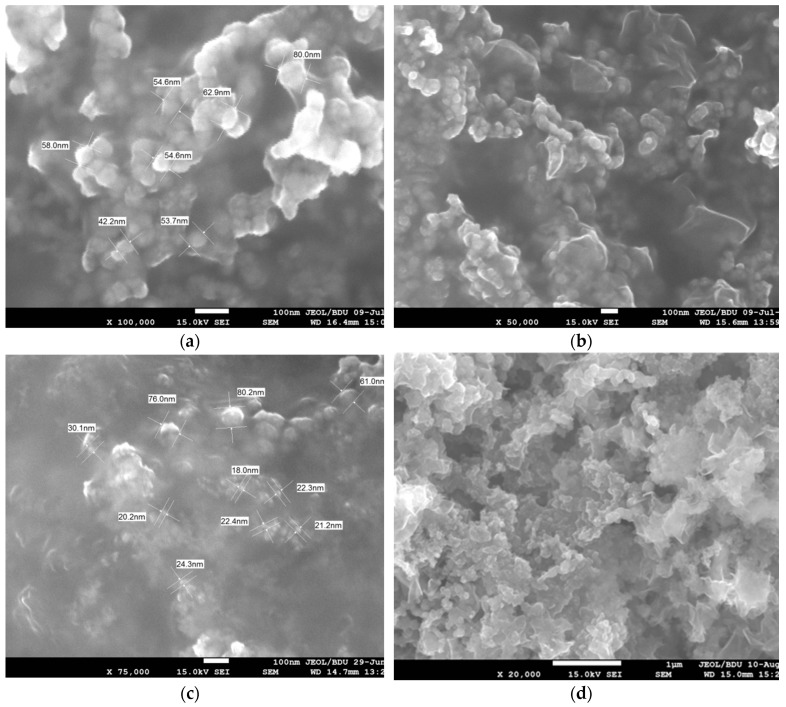
SEM images of zerovalent iron (nZVI) nanoparticles (**a**,**b**) and 0.5% Ni loading Fe/Ni bimetallic nanoparticles (**c**,**d**).

The morphologies of bimetallic Fe/Ni nanoparticles were studied by SEM. Figure 1a,b show the SEM images of freshly synthesized nZVI nanoparticles without the addition of the second metal. It can be observed that iron particles were in the form of nanospheres, which were in contact with each other and formed chains having diameters of 40–80 nm. Figure 1c,d show the SEM images of Fe/Ni nanoparticles. The particles were well dispersed and the specific surface area of the particles increased due to the joining of nickel. As shown by the SEM images, the size of the nanoparticles was significantly reduced by approximately 10–60 nm after the addition of the second metal.

**Figure 2:**
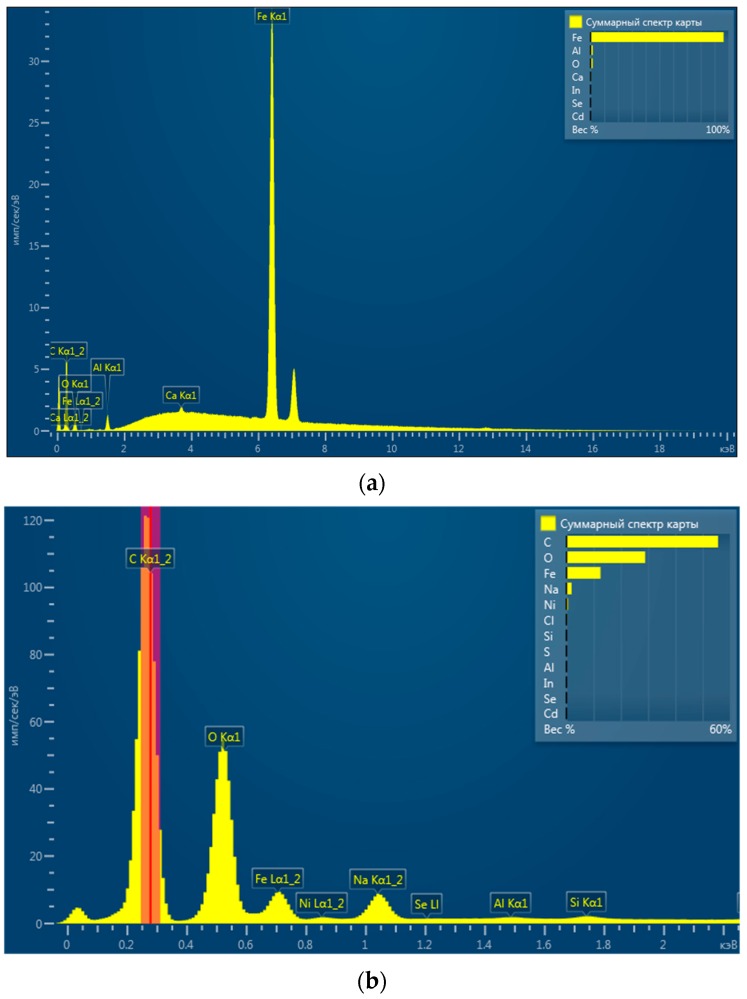
EDS spectra of nZVI nanoparticles (**a**) and 0.5% Ni loading Fe/Ni bimetallic nanoparticles (**b**).

The presence of iron and nickel in the synthesized nanoparticles was confirmed by EDS, as shown in Figure 2, where the energy-dispersive spectrum of nZVI and bimetallic nanoparticles is displayed. EDS analysis of nZVI and bimetallic nanoparticles showed peaks corresponding to Fe and Ni elements. The other elements on the EDS spectrum, such as carbon, oxygen, sodium, and aluminum, came from other sources. The presence of oxygen was associated with an oxidized layer of iron nanoparticles. Carbon and aluminum came from the substrate used to deposit the nanoparticles. Sodium was probably the residue of the sodium oleate used for the synthesis of the nanoparticles.

**Figure 3:**
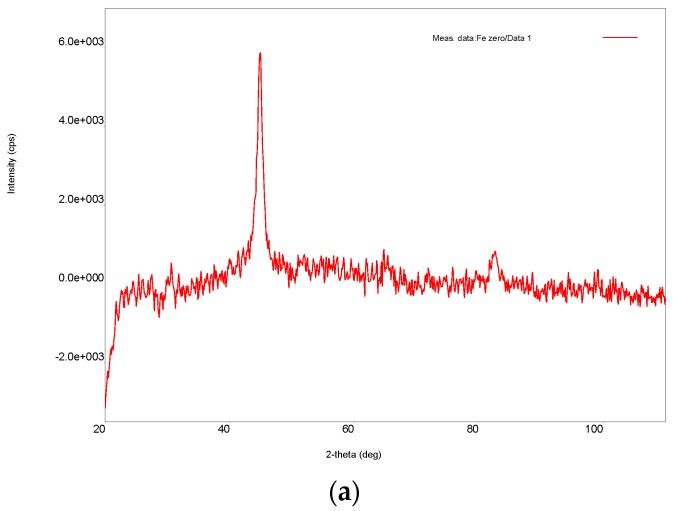

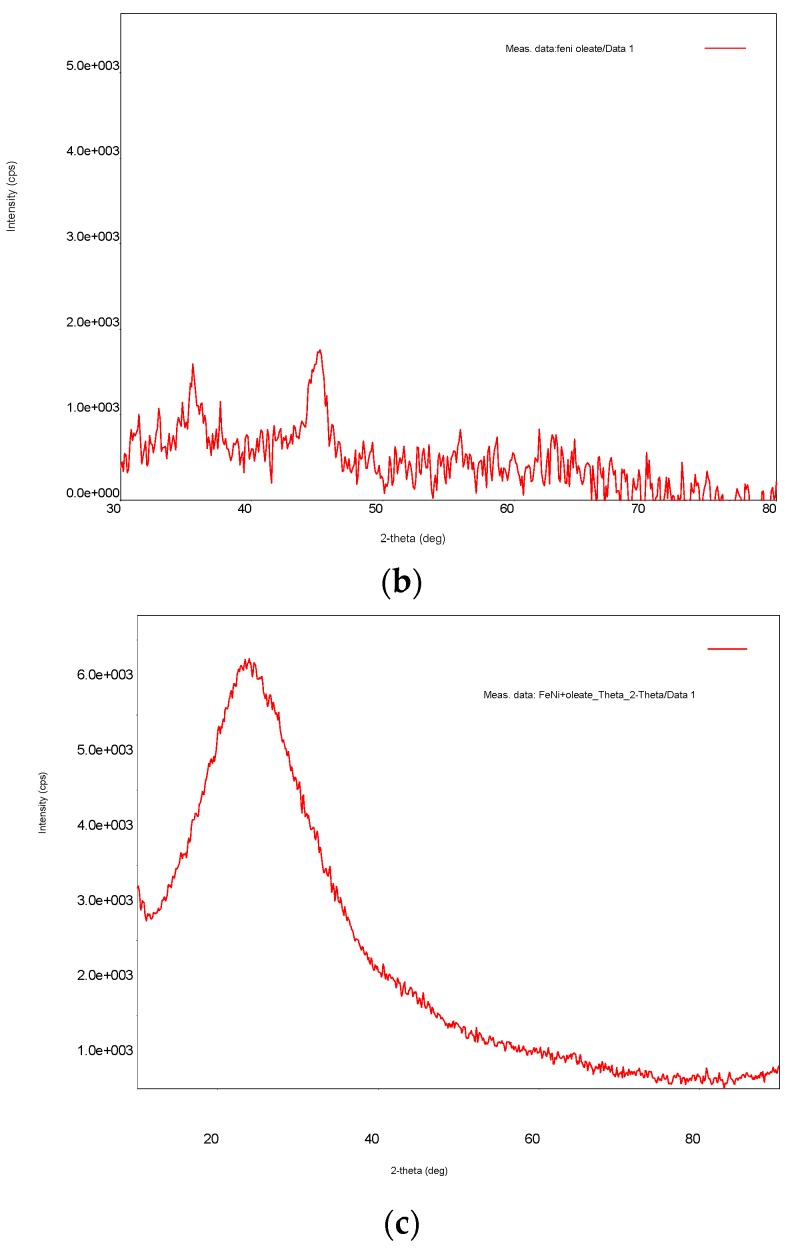
XRD of nZVI nanoparticles (**a**), 0.5% Ni loading Fe/Ni bimetallic nanoparticles without sodium oleate (**b**), and with sodium oleate (**c**).

The XRD diffraction analysis (Figure 3a–c) showed a neat and wide peak when Fe/Ni nanoparticles were synthetized in the presence of sodium oleate. This indicates that the synthesized bimetallic nanoparticles were in an amorphous phase, as reported in other studies in the literature [34,35]. In the absence of sodium oleate, nanoparticle synthesis was not successful, as shown in Figure 3b.

### 3.2. Nitrate Reduction Tests

**Figure 4:**
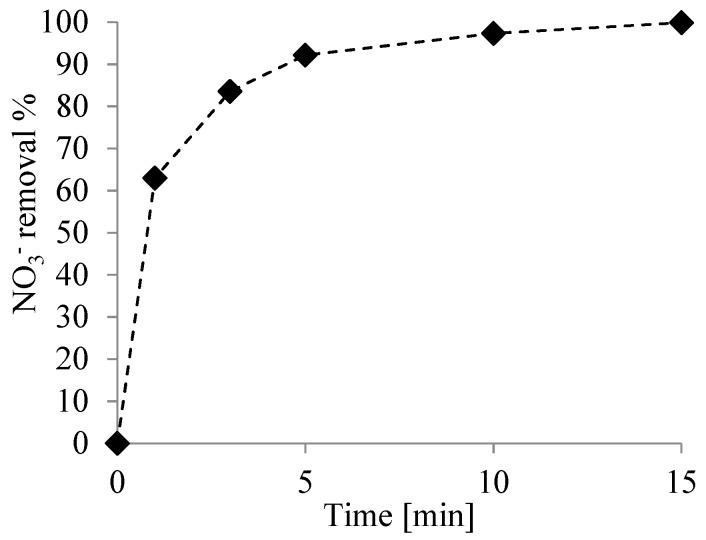
Nitrate removal. Condition: free pH, T = 298 K, 0.2 g L^−1^ bimetallic nanoparticles, and [NO^3−^] = 300 mg L^−1^.

The effect of Fe/Ni dosages on nitrate degradation at the initial nitrate concentration of 300 mg L^−1^ is shown in Figure 4. Such a nitrate concentration was substantially higher than those tested in previous studies, where a lower amount of nitrates was treated [8,27,33].

Figure 4 shows that a quick nitrate reduction occurred when 0.2 g L^−1^ of nanoparticles were added.

This result is consistent with experiments performed by other authors at lower nitrate concentrations [32,33,36]. It has been found that Ni^0^ acts as an electron donor, thus preventing iron corrosion and preserving its activity over time [33].

According to other studies, nitrate reduction by bimetallic Fe/Ni particles is a multistep reaction, involving nitrite as the intermediate product and ammonia and gaseous nitrogen as the main end-products [33,36]:

(1) $$5{\mathrm{Fe}}^{0}+2{\mathrm{NO}}_{3}^{-}+6H_{2}O\to 5{\mathrm{Fe}}^{2+}+N_{2\left(g\right)}+12{\mathrm{OH}}^{-}$$

(2) $$4{\mathrm{Fe}}^{0}+{\mathrm{NO}}_{3}^{-}+10H^{+}\to 4{\mathrm{Fe}}^{2+}+{\mathrm{NH}}_{4}^{+}+H_{2}O.$$

In the present work, at a nanoparticle dosage of 0.2 g L^−1^, an almost quantitative nitrate removal was achieved within 15 min of reaction, and the abovementioned pathway was confirmed by the mass balance performed in the tests: nitrate mostly turned into nitrogen gas and ammonia, while the nitrite concentration was negligible. In the test performed in the closed system, a residual ammonia concentration in water of 6.57 mg L^−1^ (corresponding to about 10% of the initial total nitrogen) was observed.

This result is consistent with the results of previous experiments, where Fe/Cu nanoparticles were used and the ammonia concentration was about 12% of the total nitrogen at the end of the test [37].

Furthermore, both reactions (1) and (2) involved a progressive increase of pH, causing a corresponding reduction of the nitrate removal rate [33,36].

**Figure 5:**
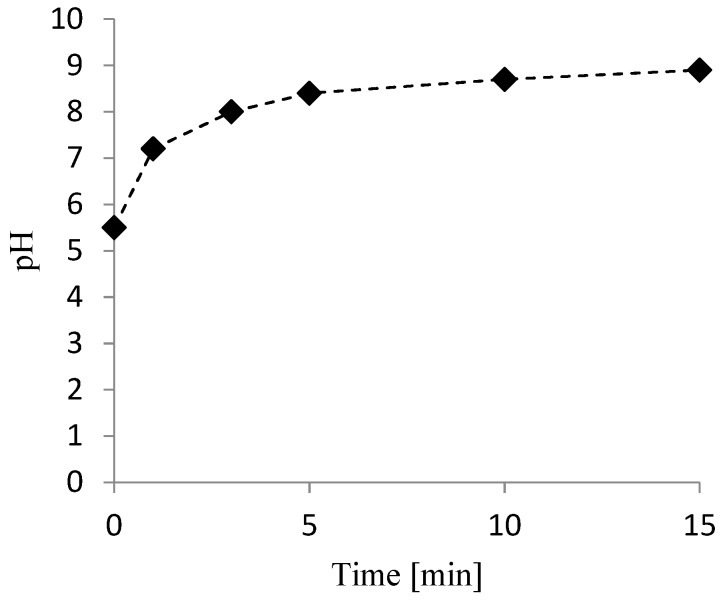
pH evolution during nitrate removal. Condition: unbuffered pH, T = 298 K, 0.2 g L^−1^ bimetallic nanoparticles, and [NO_3_^−^] = 300 mg L^−1^.

Figure 5, which reports the pH evolution over time, shows that in the test performed at 0.2 g L^−1^ of Fe/Ni nanoparticles, alkaline conditions (pH 8.4) were already reached in the first 5 min of treatment due to the development of the above-reported reactions.

**Figure 6:**
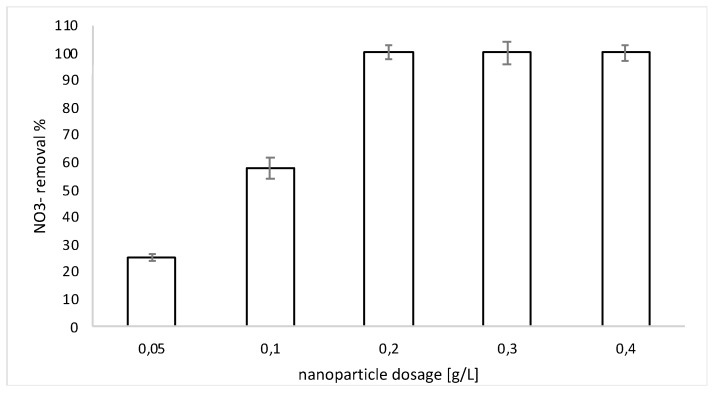
Nitrate removal at different dosages of bimetallic nanoparticles. Condition: unbuffered pH, T = 298 K, and [NO_3_^−^] = 300 mg L^−^^1^.

Figure 6 shows the results of the test performed at different nanoparticle dosages. Only at concentrations of nanoparticles higher than 0.2 g L^−1^ was it possible to achieve an almost complete removal (99.8%) of nitrate after 15 min of treatment at free pH conditions.

It was therefore clear that pH plays a key role in the effectiveness of the treatment. For this reason, additional tests were conducted at different initial pH values (3, 5, and 10) by using 0.1 g L^−1^ of nanoparticles to assess the operating conditions that maximized nitrate removal.

**Figure 7:**
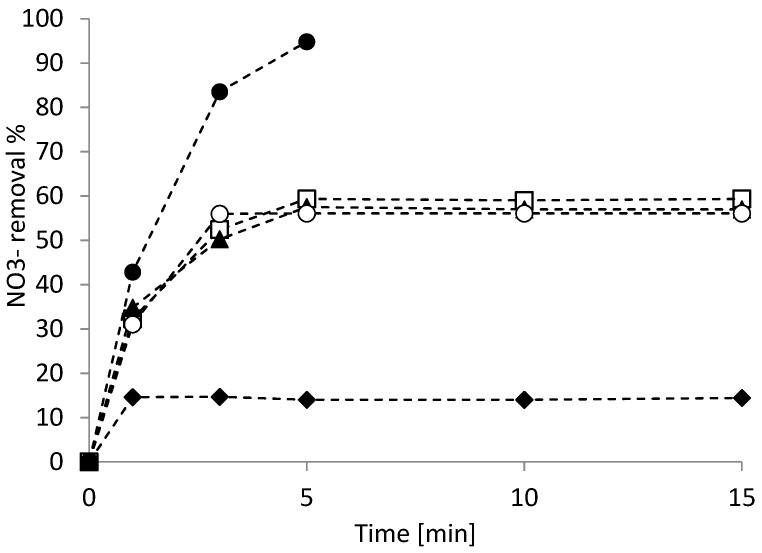
Nitrate removal at different pH conditions: unbuffered (▲); buffered at pH = 3 (●); and initial pH = 3 (□), 5 (○), and 10 (◆). Condition: T = 298 K, 0.1 g L^−1^ bimetallic nanoparticles, and [NO_3_^−^] = 300 mg L^−1^.

The results of the tests performed to assess the influence of pH on nitrate removal are reported in Figure 7.

The results shown in Figure 6 confirmed the effect of pH on nitrate removal: a pH of 10 stopped the reaction within the first minute of treatment. Regarding the tests at the initial pH of 3 and 5, in both conditions, a 60% removal was quickly reached after 3 min of treatment, which then settled down as soon as a pH value of 9 was reached, thus preventing any further nitrate removal.

The free pH test was conducted not by modifying the pH but leaving it in its normal evolution during the reaction. The solution had an approximately neutral pH and then evolved to pH 9. Also, in this case, no difference in terms of nitrate removal was observed; up to 60% of NO_3_^−^ was calculated after 3 min of treatment. This result suggests that it is sufficient to maintain pH conditions below 6 in order to guarantee good treatment efficiencies. This is in accordance with other studies, where it was reported that Fe^0^ maintained high reduction properties at low-pH conditions with rapid nitrate removal [32], and negligible NO_3_^−^ removal was noticed when the pH was higher than 6.5 [38].

To confirm that the occurrence of an alkaline pH condition stopped the reaction evolution, further tests were conducted by maintaining a fixed pH condition at 3. As shown in Figure 6, when the NO_3_^−^ removal was operated at a fixed pH 3 condition, almost complete nitrate removal (94.85%) was obtained within 5 min.

By comparing the trend corresponding to the test at a fixed pH of 3 and only the initial pH being 3, it was possible to observe that in 3 min of treatment, the alkalization of the solution already involved a strong limitation of the reaction. A removal of 83.56% was recorded when an acidic condition was ensured, and a decrease of such value at 52.42% was the result of achieving an alkaline pH (close to 9).

According to these results, the minimization of the nanoparticle concentration (0.1 g L^−1^) can be achieved by fixing the pH at 3, which results in total removal (94.85%). Different studies demonstrated nitrate reduction by using a greater amount of nanoparticles: 1.0 g [8] and 4 g L^−1^ [33].

Finally, to better achieve a comparison among the different tested conditions, efficiency evaluations of turnover number (TON) and turnover frequency (TOF) were calculated for all the tests carried out at a catalyst dosage of 0.1 g/L. A modified TON was estimated by considering the catalyst loading expressed in the molar dosage and not considering the specific surface (assuming that there is a proportionality between load and surface), as reported in Table 1.

### 3.3. Kinetic Study

**Figure 8:**
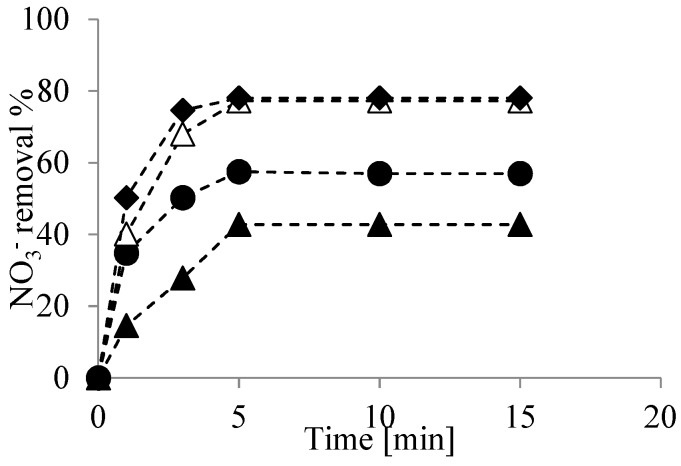
Nitrate removal at different temperature conditions: 283 (▲), 298 (●), 303 (Δ), and 313 K (♦). Condition: unbuffered pH, 0.1 g L^−1^ bimetallic nanoparticles, and [NO_3_^−^] = 300 mg L^−1^.

To study the removal kinetics, tests were conducted while maintaining the concentration of nanoparticles (0.1 g L^−1^) and unbuffered pH in order to investigate the effect of temperature on nitrate removal. The tests were performed at different temperatures (283, 298, 303, and 313 K) and the results are reported in Figure 8.

A positive effect of temperature increase was observed in terms of both nitrate removal efficiency and reaction kinetics [39]. High temperatures up to 313 K resulted in an improvement in the process, with 74% nitrate removal after 5 min of treatment at free pH with respect to the same test at 298 K. As a result, by increasing the temperature, it was possible to balance the negative effect due the alkalization, and an enhancement of NO_3_^−^ removal was observed.

Like in other works [18,40], the experimental results were then fitted using first-order kinetics (Equation (3)).

(3) $$\frac{d{\mathrm{NO}}_{3}^{-}}{dt}=-k{\mathrm{NO}}_{3}^{-}$$

**Figure 9:**
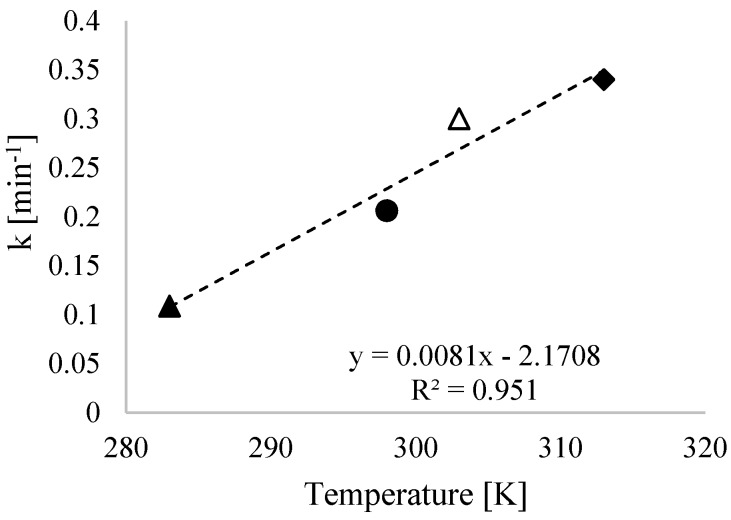
First-order kinetic constants at different temperature conditions: 283 (▲), 298 (●), 303 (Δ), and 313 K (♦). Condition: unbuffered pH, 0.1 g L^−1^ bimetallic nanoparticles, and [NO_3_^−^] = 300 mg L^−1^.

From the fitting of the experimental data (Figure 9), the calculated kinetic constants were equal to 10.88 × 10^−2^, 20.61 × 10^−2^, 30.00 × 10^−2^, and 34.00 × 10^−2^ min^−1^ for 283, 293, 303, and 313 K, respectively.

These results are to be considered positive: the presence of Ni ensured the highest removal of nitrate under milder operating conditions than those obtained in another study by only using nanoscale zerovalent iron at 348 K and a buffered neutral pH condition [41]. In such conditions, a kinetic constant of 23 × 10^−3^ min^−1^ was calculated, which is about 32% less than what was obtained in the present work at 313 K.

A further comparison with other studies in the literature also shows that, by using bimetallic nanoparticles, almost complete nitrate removal can be achieved at a lower nanoparticle dosage (0.1 g L^−1^) than those proposed thus far (from 0.5 [41] to 4 g L^−1^ [33]) and with higher kinetic constants with respect to those previously observed (26.20 × 10^−3^ [41] and 6.30 × 10^−3^ min^−1^ [33].

## 4. Conclusions

Synthesized Fe/Ni bimetallic nanoparticles (in various concentrations of 0.05, 0.1, 0.2, 0.3, and 0.4 g L^−1^) were successfully tested for nitrate removal from high-strength water. Fast nitrate removal was obtained from water at 300 mg L^−1^ of nitrates; more than 99% of nitrates were reduced within 15 min by using a concentration of nanoparticles of at least 0.2 g L^−1^. The process efficiency was found to strongly depend upon pH. Since alkaline conditions were attained during the reaction, nitrate removal mainly occurred in the first 5 min and stopped when the pH reached a value of around 8.4. Tests performed to reduce the number of nanoparticles used in the treatment showed that at 313 K, with a nanoparticle concentration of 0.1 g L^−1^ and at a buffered pH = 3, 94.85% nitrate removal was obtained. Overall, these results proved that Ni^0^ enhanced the activity of nanoscale zerovalent iron and favored the conversion of nitrates into gaseous nitrogen. As a result, chemical reduction by Fe/Ni nanoparticles can be successfully used for nitrate removal from water as an alternative to biological processes.

## Figures and Tables

**Table 1:** Turnover number (TON) and turnover frequency (TOF) calculated at a catalyst dosage of 0.1 g/L.

Test Condition				
pH	T (K)	NO_3_^−^ Removal (%)	TON	TOF (h^−1^)
unbuffered	298	57	1.59	6.37
3	298	59.34	1.66	6.63
buffered at 3	298	94.85	2.65	10.60
5	298	56.08	1.57	6.27
10	298	14.41	0.40	1.61
unbuffered	283	42.74	1.19	4.78
unbuffered	303	77.23	2.16	8.63
unbuffered	313	78.04	2.18	8.72

## References

[1] Bhatnagar A., Sillanpää M. (2011). A review of emerging adsorbents for nitrate removal from water. Chem. Eng. J..

[2] Liu Y., Majetich S.A., Tilton R.D., Sholl D.S., Lowry G.V. (2011). TCE dechlorination rates, pathways, and efficiency of nano-scale iron particles with different properties. Environ. Sci. Technol..

[3] Prakasa Rao E.V.S., Puttanna K. (2000). Nitrates, agriculture and environment. Curr. Sci..

[4] Majumdar D., Gupta N. (2000). Nitrate pollution of groundwater and associated human health disorders. Indian J. Environ. Health.

[5] Forde B.G. (2000). Nitrate transporters in plants: Structure, function and regulation. Biochim. Biophys. Acta Biomembr..

[6] Kilfoy B.A., Ward M.H., Zheng T., Holford T.R., Boyle P., Zhao P., Dai M., Leaderer B., Zhang Y. (2010). Risk of non-Hodgkin lymphoma and nitrate and nitrite from the diet in Connecticut women. Cancer Causes Control.

[7] Fewtrell L. (2004). Drinking-water nitrate, methemoglobinemia, and global burden of disease. Environ. Health Perspect..

[8] Sparis D., Mystrioti C., Xenidis A., Papassiopi N. (2013). Reduction of nitrate by copper-coated ZVI nanoparticles. Desalin. Water Treat..

[9] Schoeman J.J., Steyn A. (2003). Nitrate removal with reverse osmosis in a rural area in South Africa. Desalination.

[10] Torabian A.S., Hassani A., Abedi S.M. (2006). Comparing reverse osmosis and ion exchange methods of nitrate elimination. J. Environ. Sci. Technol..

[11] Zumft W.G. (1997). Cell biology and molecular basis of denitrification. Microbiol. Mol. Biol. Rev..

[12] Kim Y.H., Hwang E.D., Shin W.S., Choi J.H., Ha T.W., Choi S.J. (2007). Treatments of stainless steel wastewater containing a high concentration of nitrate using reverse osmosis and nanomembranes. Desalination.

[13] Choi J., Batchelor B., Won C., Chung J. (2012). Nitrate reduction by green rusts modified with trace metals. Chemosphere.

[14] Guo J., Guo P., Yu M., Sun Z., Li P., Yang T., Liu J., Zhang L. (2018). Chemical reduction of nitrate using nanoscale bimetallic iron/copper particles. Pol. J. Environ. Stud..

[15] Kumar M., Chakraborty S. (2006). Chemical denitrification of water by zero-valent magnesium powder. J. Hazard. Mater..

[16] Murphy A.P. (1991). Chemical removal of nitrate from water. Nature.

[17] Sohn K., Kang S.W., Ahn S., Woo M., Yang S.K. (2006). Fe (0) nanoparticles for nitrate reduction: Stability, reactivity, and transformation. Environ. Sci. Technol..

[18] Kim D.G., Hwang Y.H., Shin H.S., Ko S.O. (2016). Kinetics of nitrate adsorption and reduction by nano-scale zero valent iron (NZVI): Effect of ionic strength and initial pH. KSCE J. Civil Eng..

[19] Azzam A.M., El-Wakeel S.T., Mostafa B.B., El-Shahat M. (2016). Removal of Pb, Cd, Cu and Ni from aqueous solution using nano scale zero valent iron particles. J. Environ. Chem. Eng..

[20] Biterna M., Antonoglou L., Lazou E., Voutsa D. (2010). Arsenite removal from waters by zero valent iron: Batch and column tests. Chemosphere.

[21] Biterna M., Arditsoglou A., Tsikouras E., Voutsa D. (2007). Arsenate removal by zero valent iron: Batch and column tests. J. Hazard. Mater..

[22] Zeng Y., Walker H., Zhu Q. (2017). Reduction of nitrate by NaY zeolite supported Fe, Cu/Fe and Mn/Fe nanoparticles. J. Hazard. Mater..

[23] Khalil A.M., Eljamal O., Amen T.W., Sugihara Y., Matsunaga N. (2017). Optimized nano­scale zero-valent iron supported on treated activated carbon for enhanced nitrate and phosphate removal from water. Chem. Eng. J..

[24] Malakootian M., Yaghmaian K., Tahergorabi M. (2011). The efficiency of nitrate removal in drinking water using iron nano-particle: Determination of optimum conditions. J. Yazd Univ. Med. Sci..

[25] Fenglian F., Dionysios D.D., Liu L. (2017). The use of zero-valent iron for groundwater remediation and wastewater treatment. J. Hazard. Mater..

[26] Shanawar H., Sungjun B., Woojin L., Muhammad T.A., Abdulrahman A.A. (2015). Catalytic nitrate removal in continuous bimetallic Cu−Pd/nanoscale zerovalent iron system. Ind. Eng. Chem. Res..

[27] Daud M., Khan Z., Ashgar A., Danish M.I., Qazi I.A. (2015). Comparing and optimizing nitrate adsorption from aqueous solution using Fe/Pt bimetallic nanoparticles and anion exchange resins. J. Nanotechnol..

[28] Yang J., Hou B., Wang J., Tian B., Bi J., Wang N., Li X., Huang X. (2019). Nanomaterials for the removal of heavy metals from wastewater. Nanomaterials.

[29] Shi J., Long C., Li A. (2016). Selective reduction of nitrate into nitrogen using Fe-Pd bimetallic nanoparticle supported on chelating resin at near-neutral pH. Chem. Eng. J..

[30] Huang K.C., Ehrman S.H. (2007). Synthesis of iron nanoparticles via chemical reduction with palladium ion seeds. Langmuir.

[31] Yuvakkumar R., Elango V., Rajendran V., Kannan N. (2011). Preparation and characterization of zero valent iron nanoparticles. Dig. J. Nanomater. Biostruct..

[32] Kang H., Xiu Z., Chen J., Cao W., Guo Y., Li T., Jin Z. (2012). Reduction of nitrate by bimetallic Fe/Ni nanoparticles. Environ. Technol..

[33] Li P., Lin K., Fang Z., Wang K. (2017). Enhanced nitrate removal by novel bimetallic Fe/Ni nanoparticles supported on biochar. J. Clean. Prod..

[34] Wang C.Y., Chen Z.Y., Cheng B., Zhu Y.R., Liu H.J. (1999). The preparation, surface modification and characterization of metallic α-Fe nanoparticles. Mater. Sci. Eng. B Solid State Mater. Adv. Technol..

[35] Glavee G.N., Klabunde K.J., Sorensen C.M., Hadjipanayis G.C. (1995). Chemistry of borohydride reduction of iron (II) and iron (III) ions in aqueous and nonaqueous media. Formation of nanoscale Fe, FeB, and Fe2B powders. Inorg. Chem..

[36] He Y., Lin H., Dong Y., Li B., Wang L., Chu S., Liu J. (2018). Zeolite supported Fe/Ni bimetallic nanoparticles for simultaneous removal of nitrate and phosphate: Synergistic effect and mechanism. Chem. Eng. J..

[37] Muradova G., Gadjieva S., Di Palma L., Vilardi G. (2016). Nitrates removal by bimetallic nanoparticles in water. Chem. Eng. Trans..

[38] Cheng I.F., Muftikian R., Fernando Q., Korte N. (1997). Reduction of nitrate to ammonia by zero-valent iron. Chemosphere.

[39] Ji M.K., Ahn Y.T., Ali Khan M., Abou-Shanab R.A.I., Cho Y., Choi J.Y., Je Kim Y., Song H., Jeon B.H. (2011). Removal of nitrate and ammonium ions from livestock wastewater by hybrid systems composed of zero-valent iron and adsorbents. Environ. Technol..

[40] Hwang Y.H., Kim D.G., Ahn Y.T., Moon C.M., Shin H.S. (2010). Fate of nitrogen species in nitrate reduction by nanoscale zero valent iron and characterization of the reaction kinetics. Water Sci. Technol..

[41] Ahn S.C., Oh S.Y., Cha D.K. (2008). Enhanced reduction of nitrate by zero-valent iron at elevated temperatures. J. Hazard. Mater..

